# Efficient Luminescence of Sr_2_Si_5_N_8_:Eu^2+^ nanophosphor and its film applications to LED and Solar cell as a downconverter

**DOI:** 10.1038/s41598-020-58469-7

**Published:** 2020-01-30

**Authors:** Taewook Kang, Sunghoon Lee, Taehoon Kim, Jongsu Kim

**Affiliations:** 1 Interdisciplinary Program of LED Convergence, Pukyong National University, Busan, 48513 Republic of Korea; 2 Cell Bio Korea Co. Ltd., Seoul, 07547 Republic of Korea; 3 Ujin materials, Busan, 48547 Republic of Korea; 4 Department of Display and Science Engineering, Pukyong National University, Busan, 48513 Republic of Korea

**Keywords:** Nanoparticles, Nanoparticles

## Abstract

Here we present the synthesis of the efficient nanophosphor Sr_2_Si_5_N_8_:Eu^2+^ (D_50_ = 144 nm) by a simple milling approach, its strong Rayleigh scattering, and its film applications to white LED and silicon solar cell as a downshifting medium. The final nanophosphor product showed the quantum efficiency comparable to the bulk phosphor which is, to our knowledge, the highest record of nitride nanophosphors. Especially the nanophosphor showed the more tail emission at the shorter-wavelength side of the emission spectrum and the faster thermal quenching with the more spectral broadening along with the temperature due to Rayleigh scattering. Also the lowering in the excitation spectrum was observed due to lower absorbance. Finally, the nanophosphor-dispersed polyvinyl alcohol (PVA) film was made, and its applications to white LED and silicon solar cell as a downshifting medium demonstrated that it gave the high color rendering property in white LED in spite of still lower luminous efficiency, and it caused the increase in efficiency of silicon solar cell.

## Introduction

As the wide possibilities for nanotechnology in chemistry, physics, biology, and materials science, intensive efforts have been paid to the development of new techniques for synthesis and characterization of nanophosphors^1,2^. In principle, nanophosphors can be obtained by either top-down process of the bulk phosphor like milling process or bottom-up process like a low-temperature synthesis method.

As a simple example of the top-down, some nanophosphors are obtained through a high-energy milling of large phosphors synthesized by a high-temperature solid-state reaction. The bottom-up method is reported to be an effective technique to control the final phosphor size through solvothermal or microwave-assisted reactions by adding some amounts of surfactants, or chelating reagents. Up to now a number of various synthesis methods has been demonstrated to control size distribution and morphology of nanophosphors; laser ablation^3^, microemulsion route^4^, template-directed synthesis^5^, and single-precursor thermal decomposition^6^.

A mechanical milling process is a cost-effective mass production technique, which is mostly realized with some amount of alumina and zirconia balls with high-speed rotation energy, such as compression, shear and impact^7^. The size distribution the final nanophosphors is controlled by some parameters, including (1) the type of the mills such as general ball mill, planetary mill, attrition mill, and jet mill, (2) the nature of milling media such as glass, steel, alumina, and zirconia, (3) the ball-to-powder-to-solvent ratios, (4) the total volume ratios, (5) the rotation speed, and (6) the ball size^8,9^. The mechanical milling method has some advantages over other methods: simple and cheap experimental setup, easy and eco-friendly process, and mass production possible. Especially, the industrial planetary mills with continuous operation have been well developed for mass production with a rate of several tons per hour. On the other hand, it has some serious problems as a direct milling process of bulk phosphor; it may produce the non-spherical nanophosphors with a poor crystallinity with a large number of surface defects, or by-products (impurities) reformed by high impact energy, which can significantly deteriorate the optical properties due to their light trapping. To minimize these negative effects, the post annealing or the post acid washing procedure should be introduced.

One of challenging nanophosphors is the nitride phosphor which has been utilized as a red phosphor in white light emitting diodes^10^ and as a color shifting material in solar cell^11,12^. This is the reason why the nitride phosphors structurally built up on highly condensed framework with very strong covalent bonds of M-N or X-N, which are synthesized with a mixture of MN_x_ (M = Ca, Sr, Ba, and Eu), and XN_4_ (X = Si or Al) at the temperature of at least more than 1600 °C under the high pressure of nitrogen gas. For examples, there are two commercially available Eu^2+^-doped M_2_Si_5_N_8_ (M = Ca, Sr, Ba), and Eu-doped MAlSiN_3_ (M = Ca, Sr, Ba) due to their excellent photoluminescence properties, such as broad excitation band, tunable emission, and high quantum efficiency.

By this nature of very rigid framework synthesized at extremely ultimate synthesis conditions, the development of nitride phosphors is very challenging, and as a consequence more effort has been directed toward the new synthesis method for the nanophosphor. There are few recent works on the red nanophosphors (~200 nm in spherical shape) of M_2_Si_5_N_8_:Eu^2+^ prepared by a one-pot reaction of metal amides and nanocrystalline silicon at rather low synthesis temperature (~1400 °C)^13^. Its reported efficiency (80%) was comparable to that from a conventional high-temperature process. However, the preparation of metal amide precursors necessitates usage of closed reaction system with supercritical NH3 hampering upscaling of these approaches to large-scale processing. Recently, we have reported the high-energy milling approach to oxynitride (yellow EuSi_2_O_2_N_2_)^14^ and oxide (yellow Y_3_Al_5_O_12_:Ce)^15^ nanophosphors and the influence of milling process on the crystal shape with forming sharp edges and optical property with the luminous efficiency comparable to the bulk phosphor.

In this work, we present the synthesis of the efficient nanophosphor Sr_2_Si_5_N_8_:Eu^2+^ by a simple milling approach. The oxynitrization of nitride surface by a reaction with water solute during a milling process was observed. Its influence on the crystal shape with a mean size of 150 nm without sharp edges was investigated. Moreover, the final sphere-like nanophosphor product showed the quantum efficiency comparable to the bulk phosphor as well as its well dispersion in water after removing the formed surface oxynitride with nitric acid. Especially the nanophosphor showed the more tail emission at the shorter-wavelength side of the emission spectrum, and the lowering in the excitation spectrum, compared with the bulk phosphor. Also, its emission was more thermally quenched with the temperature than that of the bulk. Finally, the polyvinyl alcohol (PVA) film was made from the nanophosphor colloidal, and it was applied to the remote phosphor-type white LED and silicon solar cell as a downconversion layer.

## Results and Discussion

**Figure 1:**
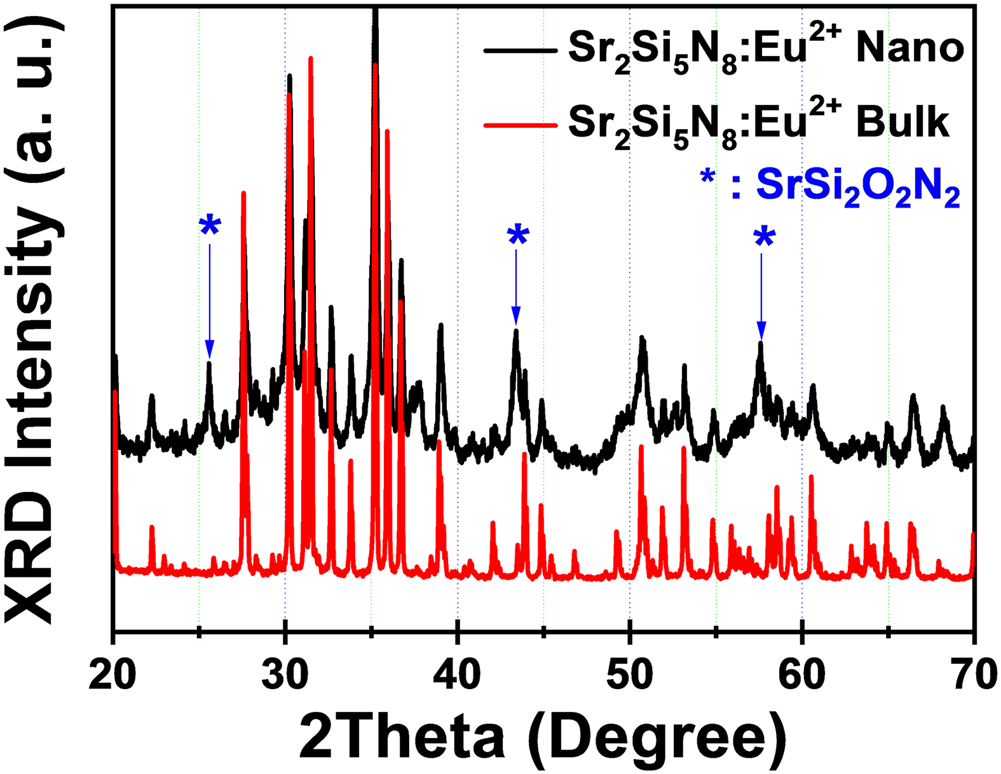
XRD pattern of Sr_2_Si_5_N_8_:Eu^2+^ bulk phosphor and weak peaks from SrSi_2_O_2_N_2_ formed during the high-energy bead milling process in nanophosphor.

Figure 1 displays the XRD pattern of the bulk phosphor fired at 1800 °C for 12 hours under a 0.5 MPa N_2_ pressure. All of the peaks are indexed as the Sr_2_Si_5_N_8_ phase (JCPDS: 85–0101), which is the orthorhombic crystal system and the space group of Pmn21 with Z = 2, a = 571.00 pm, b = 682.20 pm, and c = 934.10 pm^16,17^. As ideal Sr_2_Si_5_N_8_ and Eu_2_Si_5_N_8_ are isostructural with a nearly same ionic size of Eu^2+^ (130 pm) and Sr^2+^ (127 pm), the dopant Eu^2+^ ions can perfectly be substituted on Sr^2+^ sites in a form of solid solution. It is known that Sr^2+^ ions are caged into the channels surrounded by Si_6_N_6_ rings along the [100] orientation. It has two different kinds of Sr^2+^ sites; 8-coordinated SrI and 10-coordinated SrII sites which can be equally doped with Eu^2+^ ions. The average distance of SrI–N (286.5 pm) is shorter than that of SrII–N (292.8 pm), and thus the Eu^2+^ ions on the SrI sites is expected to be more compressed by the tighter surrounding so as to experience the higher crystal field than those on the SrII site. Furthermore, their different crystal field strength may cause the different photoluminescent properties, as shown in the later section.

As shown in Fig. 1(b), weak characteristic peaks from SrSi_2_O_2_N_2_ formed during the high-energy bead milling process were observed, and the more background noises along with broadening were shown in XRD pattern. It is considered that the SrSi_2_O_2_N_2_ phase results from water-driven oxidation of stressed or milled Sr_2_Si_5_N_8_ surface at high temperature (~700 °C) heated by a high-energy impact during milling process. The background noises are also attributed to SrSi_2_O_2_N_2_ amorphous phase. The new several weak peaks (indexed as *) comes from crystalline SrSi_2_O_2_N_2_ phase, which was a little removed by acid washing so as to enhance the luminescent intensity, as shown in the later section. The XRD pattern broadening can be described by the effect of crystal size and imperfections, that is, Sherrer’s law: mean particle size ~K·λ/βcosθ where K is a dimensionless shape factor with a value close to unity; λ is the X-ray wavelength; β is the line broadening; θ is the Bragg angle. Calculated particle sizes were consistent with the following values from FE-SEM and PSA: D_50_ = 144 nm.

**Figure 2:**
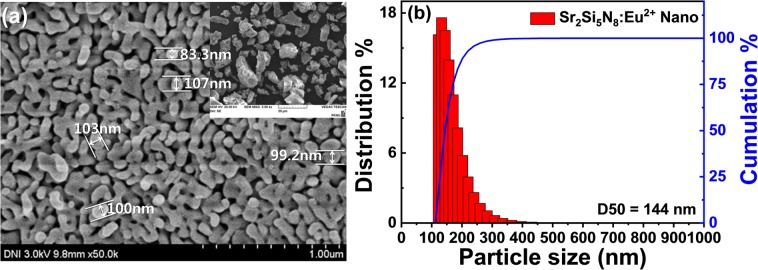
SEM image of Sr_2_Si_5_N_8_:Eu^2+^ (**a**) of nanophosphor (the bulk image in the inset), and (**b**) PSA spectrum of Sr_2_Si_5_N_8_:Eu^2+^ nanophosphor.

Figure 2 shows particle size analysis (PSA) data and FE-SEM image of the nanophosphor. Particle size distribution showed in 100–200 nm range and their mean particle sizes (D_50_) was 144 nm. It is notable that the nanophosphor showed the spherical morphology, even though it was prepared through a high-energy planetary milling and expected to be irregular and edged shape. It can be explained by grounding and oxynitrization effects of the sharped edge of the nitride surface during the high-energy bead milling.

**Figure 3:**
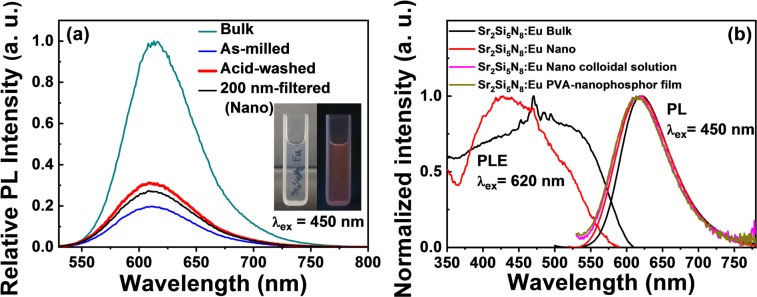
The comparison of relative PL intensities for each process (**a**), and PL and PLE spectra of Sr_2_Si_5_N_8_:Eu^2+^ bulk/nanophosphors (**b**). The inset images show the colloidal solution with a 0.05 wt.% concentration (1.8% haze in penetration depth of 5 mm) without (left) and with (right) UV lamp.

Figure 3(a) shows PL (excited by 450 nm light) spectra of Sr_2_Si_5_N_8_:Eu^2+^ nanophosphor and the bulk reference phosphor to instigate the effect of the post treatment of the as-milled nanophosphor. The PL intensity of the as-milled sample was decreased up to 20% of that of the bulk phosphor due to some defects such as oxynitride surface defects and internal crystal defects, and its PL intensity was increased up to 30% after a nitric acid washing due to the removal of some surface defects, as confirmed in Fig. 1(a). Additionally, the acid washed nanophosphor was well drifted and dispersed into water due the acid modification of surface, but the colloidal water solution with a concentration of 0.05 wt.% in pure water showed a little high haze of 1.8% in the penetration depth of 5 mm due to a large scattering effect resulting from the very high refractive index of 2.55 of the nitride material, as confirmed in the inset pictures by naked eyes. Subsequently the filtered nanophosphor below 200 nm showed the slight decrease in PL intensity due to the removal of the big sized particles with a relatively high brightness. The final nanophosphor showed the size distribution with D_50_ of 144 nm and 27% of the PL intensity of the bulk phosphor.

The most interesting thing is that the internal quantum efficiency of the nanophosphor (~61%) is comparable to that of the bulk phosphor (74%), while the external quantum efficiency of the nanophosphor (~20%) is inferior to that of the bulk phosphor (64%) due to the very lower absorption of the nanophosphor. The highest reported quantum efficiency of Sr_2_Si_5_N_8_:Eu^2+^ nanophosphors prepared at low temperature (~1400 °C) by thermal decomposition of a single source precursor mixture is about 80% with respect to the bulk phosphor, where our relative result to the bulk (61/74) is 82%, which is higher than the previous one^13^. As a reference, the one highest reported quantum efficiency of YAG:Ce^3+^ nanophosphors passivated with an intercalated layered alumina synthesized through a solvothermal method is about 57%^18^, and the other one of YAG:Ce nanophosphors made by the combustion synthesis was reported to be about 54%^19^.

Figure 3(b) shows PLE (monitored at 620 nm) and PL (excited by 450 nm light) spectra of Sr_2_Si_5_N_8_:Eu^2+^ nanophosphor and the bulk reference phosphor. Both samples showed broad red spectra: λ(SrI + SrII) = 615 nm, Δλ(SrI + SrII) = 70 nm for the bulk, and λ(Sr1 + Sr2) = 607 nm and Δλ(Sr1 + Sr2) = 78 nm for the nanophosphor. They are originated from a close overlap of 4f-5d transition peaks from two crystallographically different Sr sites: smaller 8-coordinated Sr1 and bigger smaller 10-coordinated Sr2 sites. Therefore, Eu^2+^ ions locating at the SrI sites is considered to experience stronger crystal field strength than those occupying the SrII site^17,20^. The stronger crystal field strength of Eu^2+^ on SrI site is known to lead to the longer-wavelength emission peak.

Especially, in the reflective photoluminescent mode with powder pellet (like a dense thick film), the shorter-wavelength side of PL spectrum of the nanophosphor dominates more than the bulk phosphor. It can be understood in terms of Rayleigh scattering effect, which applies to the case when the scattering particle is 10 times smaller than wavelength^21^. Our nanophosphor meets these criteria: some nanoparticles with a particle size of 62 nm < the peak wavelength of 620 nm/10. According to the fact that Rayleigh scattering intensity is proportional to 1/wavelength^4^, the shorter-wavelength side of PL spectrum is more dominated in the light scattering than the longer side, like the reason why the sky is blue. Moreover, in the transmissive photoluminescent measurement with the nanophosphor-dispersed PVA film (5 wt.%) and the colloidal solution (0.05 wt.%) (like a dilute thin film), this effect gets more prominent due to the loner penetration depth compared with that in the reflective mode.

The PLE longer-wavelength side corresponds to the excitation from 4f to the 5d level, which decreased in the nanophosphor. The lowering of excitation efficiency in longer-wavelength side can be understood in terms of the lowering of absorption rate with increasing wavelength in the case of smaller particle size than the wavelength^21^. The narrowing of PLE spectrum can be explained by weaker phonon coupling with excitation lights. The nanophosphors have lower crystallinity causing to quenching lattice vibrations so as to generate less phonons. Hence, the phonon coupling with excitation lights can be reduced, so that their PLE spectrum is narrowed, and thus the quenching of PLE intensity at longer-wavelength side for the nanophosphors is significant.

It is known that the excitation spectrum results from the product of absorbance and quantum efficiency. Assuming the similar quantum efficiencies (actually, 61% for the nanophosphor, and 74% for the bulk), it is confirmed from Fig. 2 that the excitation intensity of the nanophosphor is lower than the bulk phosphor, and the difference is increased with the wavelength longer. It indicates that the absorption rate of the nanophosphor drops more rapidly at longer wavelengths than the bulk phosphor.

**Figure 4:**
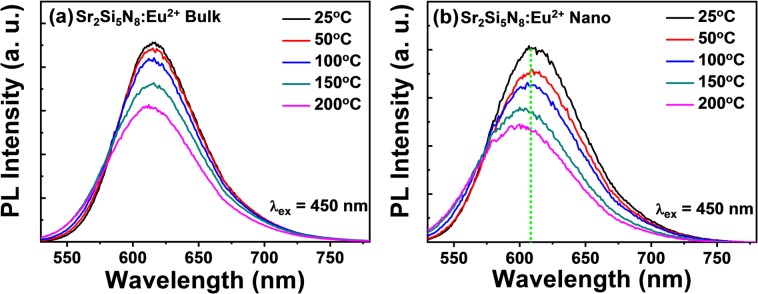
Temperature-dependent emission spectra of the nanophosphor (**a**) and the bulk phosphor (**b**).

Figure 4 displays temperature-dependent PL spectra of the nanophosphor and the bulk reference phosphor in the reflective PL measurement mode with dry powder pellets. It is generally known that as the temperature rises, the PL spectra for the bulk phosphor are gradually decreased in its intensity with a slight broadening of its peak width and a slight blueshifting of its peak position^14,15^. The thermal quenching behavior is attributed to the non-radiative release of excited electrons via the electron-phonon interaction^22^ or the thermal ionization^23^.

For our nanophosphor, the stronger thermal quenching behavior was observed compared with the bulk in reflective measurement mode, while the transmissive measurement is limited at the temperature of 100 °C where the PVA film is damaged and the water in solution evaporated. It is estimated that the higher temperature dependence of PL spectrum is a result of the stronger electron-phonon coupling effect via the assistance of more surface defects in the nanophosphor. In particular, the larger spectral shift was observed in the reflective mode with the dense dried nanophosphor pellet, compared with the spectral shift of bulk phosphor. It can be understood in terms of Rayleigh scattering theory. The approximate amount of Rayleigh scattering (I_sc_) in visible wavelengths (400 nm–700 nm) with temperature (T) is given when the particle size is 10 times smaller than the wavelength^21^;

I_sc_ ~ n^8^·T/λ^4^

where n = refractive index, λ = wavelength, and T = temperature. It is due to the microscopic variation of density and refractive index. The Sr_2_Si_5_N_8_:Eu nitride phosphor has a higher n value of 2.55, indicating its scattering effect is more significant than other oxide phosphor (n = 1.82 for YAG). Moreover, the nanophosphor has a larger surface-to-volume ratio and thus it is more sensitive to a thermal stress. In this context, the nitride nanophosphor suffers from the more significant Rayleigh scattering with temperature. As a result, our nanophosphor showed a larger Rayleigh scattering and thus the larger spectral shift than the bulk which is insensitive to the Rayleigh scattering.

**Figure 5:**
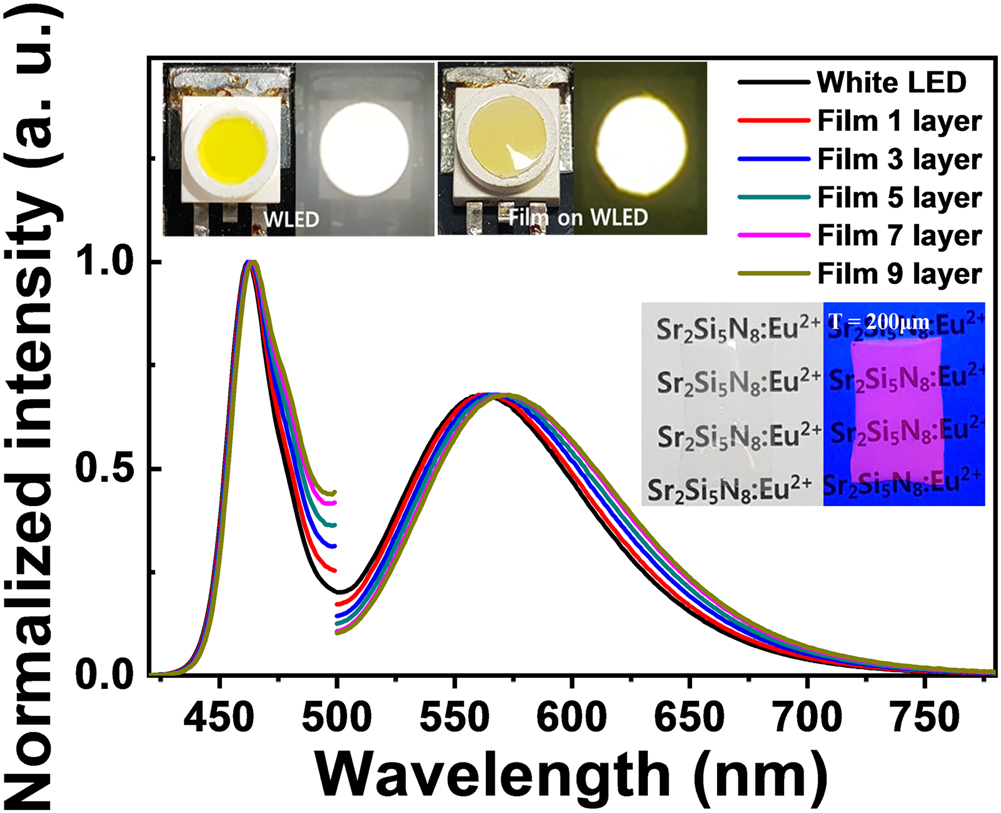
EL emission spectra of the blue-yellow based white LEDs with increasing the thickness of PVA-nanophosphor film (the number of the film layer). The top pictures show a conventional white LED (left) and a remote-type white LED (right). The bottom pictures show the PVA-nanophosphor film with 200 μm thick without (left) and under UV lamp (right).

Figure 5 shows EL emission spectra of the blue-yellow based white LEDs with increasing the thickness of PVA-nanophosphor film (the number of the film layer) in order to enhance the color rendering property. As seen in the inset image of PVA film (5 wt.%) with thick 200 μm, the nanophosphor film looks transparent with a lower haze of less than 1%. The color temperature of 5,000 K ± 500 K was fixed by adjusting the electrical power ~ the optical power of blue light as an excitation source. As the thickness of PVA-nanophosphor film increases, the color temperature was slightly changed from 5368 K to 4666 K. The luminous efficacy was exponentially decreased with increasing the number of film layer from 1 to 9; 300 lm/W_opt_ at one layer to 28 lm/W_opt_ at 9 layers. This significant decrease can be described by the reabsorption loss due to the internal efficiency of less than unity (~0.6). The relatively huge number of nanophosphors in the conversion film can cause very strong Rayleigh scattering and thus some portion of scattered lights would repeat the reabsorption process. As a result, the luminous efficacy of thee fabricated white LED was exponentially decreased. Nevertheless, it should be noted that the general color rendering index (Ra) was linearly increased by an interval of 1 from Ra = 65 for white LED without the nanophosphor film to Ra = 71 for with the number of 9 layers. It is attributed to the richness in the red color with increasing the number of film layer, like the red sky before the sunset.

**Figure 6:**
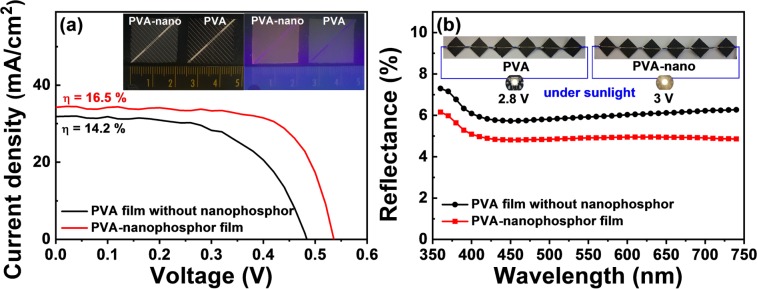
(**a**) Current-voltage curves and (**b**) reflectance of the polysilicon solar cells with and without the nanophosphor dispersed in PVA film under solar simulator and spectral reflectance measurement system. The pictures show the nanophosphor-coated and undoated film on silicon solar cell under UV lamp, connection with one white LED and the series of 6 nanophosphor-coated and uncoated solar cells.

Figure 6 shows the measured current-voltage curves of the polysilicon solar cells with and without the nanophosphor dispersed in PVA film under solar simulator (irradiated area of 1 cm^2^ by AM1.5). It has been reported that the efficiency gain in some solar cells is relatively small, even for an idealized spectral shifting material^11^. As an example, the Sr_2_Si_5_N_8_:Eu^2+^ bulk phosphor with a refractive index of 2.55, which has an absorption edge at 600 nm and an efficient red emission around 620 nm, was evaluated as an idealized spectral shifting material^12^; from the calculation, only the polycrystalline silicon solar cell of other silicon solar cells would benefit from the ideal conversion layer with a little positive efficiency gain of +0.4%. This slight increase can easily be explained by a combination of several reasons: (1) very small efficiency gain (~+1.2% for polysilicon type) for an ideal conversion layer, (2) lower quantum efficiency of the phosphor conversion (<90%), (3) scattering loss of the normal-incident absorbed light, and (4) the conversion loss in the absorption tail at the 550–600 nm region with the internal quantum efficiency of silicon solar cells of 100%. Among these loss factors, the absorption range in the nanophosphor was optimized to be in coincidence with the ideal one (the absorption edges at 541 nm and 565 nm for polysilicon and amorphous silicon, respectively). Therefore, it is expected to result in the more improvement in the efficiency gain. According to the previous work^12^, the recalculation with the modified absorption range of the nanophosphor showed the efficiency gain of about +1.0% for both polysilicon and amorphous silicon cells.

The actual application of PVA-nanophosphor film (5 wt.%) as a spectral shifting layer to the polysilicon solar cell (20 mm × 20 mm in solar cell size) caused the enhancement in the solar cell efficiency (η) as compared to a transparent PVA layer without the nanophosphor; from 14.2% to 16.5%. It is lucky that this efficiency enhancement (Δ = +2.3%) under the maximum current density of 34.5 mA/cm^2^ is considerably higher than the maximum estimate value from theoretical calculation (Δ = +1.2%) under the maximum current density of 32.0 mA/cm^2^, as shown in Fig. 6(a). It may be attributed to the increase in the transmittance and the absorbance of the nano-coated film on solar cell. We did estimate the transmittance by measuring the reflectance before and after nanophosphor coating. Both look indistinguishable in the appearance by naked eyes, but the nano-coated one glows under UV lamp. In detail, the reflectance values of 4.8% and 5.7% at 450 nm and 4.9% and 6.1% at 620 nm was measured for nano-coated and uncoated ones, respectively, as shown in Fig. 6(b). In other words, the reflectance of the nano-coated one decreases by about −1% i.e., either the transmittance or the absorbance of nano-coated one increases by about +1%.

In addition, we did the connection between these two applications. One white LED (V_th_ = 2.6 V) was connected to the series of 6 solar cells (V_on_ = 0.5 V per one cell). The voltage of nanophosphor-coated solar module is about 0.2 V higher than that of uncoated solar module. The higher brightness was observed with naked eyes under general lighting lamp in the white LED connected to the uniformly nanophosphor-coated solar module compared with the uncoated one, as shown in Fig. 6(b).

In summary, the efficient Sr_2_Si_5_N_8_:Eu^2+^ nitride nanophosphor was made through a planetary milling method. The round-shaped nanocrystals with a mean size of 144 nm without sharp edges were obtained. They showed the internal quantum efficiency comparable to the bulk phosphor which is, to our knowledge, the highest record of nitride nanophosphor (61%). They showed their unique emission and excitation spectra, which can be explained by Rayleigh scattering effect; (1) the lowering at longer wavelength side of excitation spectrum due to lowering of absorption rate with wavelength, (2) the enhancement at shorter wavelength side of emission spectrum, and (3) the faster thermal quenching with a larger spectral variation. They were well dispersed in water after acid washing like colloidal solution, from which the nanophosphor-PVA film was formed. The PVA film was over-coated on white LED and silicon solar cell, which showed the increase in a color rendering index (Ra 65 → 71) in white LED in spite of still lower luminous efficiency, and gave an information on positive variation in efficiency of solar cell (14.2% to 16.5%) as a wavelength shifting medium, which is consistent with the efficiency enhancement from theoretical calculation with a modification of absorption band edge of the nanophosphor.

## Methods

The bulk phosphor Sr_2_Si_5_N_8_:Eu^2+^ was prepared by a solid-state reaction of SrH_2_, Si_3_N_4_, and Eu_2_O_3_ with the ratio of 2:1:0.05 as starting materials. The concentration of Eu^2+^ was 5 mol % with respect to Sr^2+^. The powder mixture was conducted under a continuously purified nitrogen atmosphere in a glove box. The powder mixture was fired at 1800 °C for 12 h under a 0.5 MPa N_2_ using a gas-pressure sintering furnace with a graphite heater. The reaction product was grinded into a fine powder and subjected to an acid leaching in order to eliminate the reaction byproducts. After leaching the sediment was rinsed with warm distilled water and dried in a vacuum oven at 80 °C for 2 h. The final phosphor product showed efficient red emission with a peak of 620 nm and a half width of 80 nm as well as the acicular crystal morphology (high aspect ratio) with a mean size of 10 um, which are identical to the commercial red Sr_2_Si_5_N_8_:Eu phosphor. Subsequently the nanophosphors were prepared through a high-energy planetary milling of the bulk phosphor. For nanomilling, the bulk Sr_2_Si_5_N_8_:Eu^2+^ phosphors were mixed with deionized water solvent without any dispersant agent together with spherical ZrO_2_ beads with diameters of 800 and 300 μm with phosphor-ball-water weight ratio of 1:1:1. The mixtures were milled to the nanoscale with 10 sets of 3 minute 500 rpm rotation and 10 minutes break to prevent heating by a planetary miller (FRITSCH, Planetary Mono Mill). The intermediate nanoparticle was obtained by acid washing with 6% nitric acid for 1 hour, and rinsing 2 times. The final nanophosphor was obtained by filtering with a 200 nm syringe filtration, which was well dispersed with a concentration of 0.05 wt.% in pure water without any surfactants. The colloidal water solution was added with 10 wt.% of polyvinyl alcohol (PVA). The PVA solution was solution-casted for 200 μm thick film for white LED on slide glass, and spin-coated for 20 μm thin film for silicon solar cell. Finally, they were naturally dried for 24 hours. The obtained nanophosphor-PVA films has a little haze (less than 1% for nanophosphor concentration of 5 wt.%). The multilayer of 200 μm thick films was put on a blue LED for a required white balance. The 20 μm thin film on polysilicon solar cell (ExcelTON III, 6” momo-crystalline, ETS6-2100, maximum current density of 37 mA/cm^2^, and efficiency of 21% for non-encapsulated cell) was irradiated by solar simulator (Peccell technologies, PEC-L01). The reflectance was measured by Spectral Reflectance Measurement System (CM-3700d, Konica Minolta).
